# Association between arthritis score at the onset of the disease and long-term locomotor outcome in adjuvant-induced arthritis in rats

**DOI:** 10.1186/s13075-015-0700-8

**Published:** 2015-07-17

**Authors:** Claude Mossiat, Davy Laroche, Clément Prati, Thierry Pozzo, Céline Demougeot, Christine Marie

**Affiliations:** INSERM U1093, University Bourgogne Franche-Comté, F-21000 Dijon, France; EA4267, FHU INCREASE, University Bourgogne Franche-Comté, F-25000 Besançon, France; CHRU, Dijon, France; CHRU, Besançon, France; INSERM U 1093 Cognition, Action et Plasticité Sensorimotrice, 7 boulevard Jeanne d’Arc, BP 87900, 21000 Dijon, France

## Abstract

**Introduction:**

To investigate the connection between the intensity of initial symptoms of inflammation and locomotor outcome in rheumatoid arthritis, we examined the relationship between long-term locomotor abnormalities and signs of inflammation at the onset of the disease in adjuvant-induced arthritis (AIA) in rats.

**Methods:**

The arthritis score and hind-paw diameter were followed from immunization to day 195 (~7 months). At this time, locomotion was recorded during forced treadmill walking using 3D motion technology before radiographic scoring of hind limb joint damage. Many locomotor parameters were analyzed including time and length parameters, limbs kinematics, lateral paw position at toe off, maximal hind-paw elevation and posture. Ankle mobility was assessed from range of motion (ROM) of the joint during locomotion. Experiments were run in AIA (n = 18) and age-matched non-AIA rats (n = 8).

**Results:**

All AIA rats exhibited signs of inflammation at day 14 with a peak of inflammatory symptoms at day 22 post-immunization. After the first episode of inflammation, 83 % of AIA rats demonstrated recurrent disease (from week 6 to week 23). The frequency of inflammatory episodes (1 to 5) was not linked to the arthritis score at day 22. At day 195 post-immunization, AIA rats showed significantly impaired locomotion and radiographic lesions as compared to control rats. Significant relationships were observed between most locomotion-related parameters and concurrent ROM of ankle, which correlated negatively with the radiographic score. ROM of ankle at day 195 correlated negatively with both the arthritis score and hind-paw diameter measured at day 14, 22 and 30 post-immunization.

**Conclusion:**

Decreased ankle mobility can be considered a driver of locomotion impairment in AIA. In this model, the severity of the initial inflammatory symptoms had a good prognostic value for long-term locomotor outcome.

## Introduction

Rheumatoid arthritis (RA) is a chronic inflammatory autoimmune disease that affects about 1 % of the general population in Western countries. RA is characterized by symmetric joint involvement that can range from a monoarticular to a highly polyarticular pattern and joint damage that can span from mild cartilage degradation to progressive erosive disease of juxta-articular bone. Therefore, although RA is still regarded as a single disorder, the impact of RA on motor disability and decreased quality of life varies considerably among individuals. The decline in physical activities results in part from walking impairment but it is still unclear whether locomotor alterations are due to pain, limited range of motion (ROM) in the joint, loss of functional balance or muscle loss [1–4]. Currently, there is no reliable (either biological or clinical) prognostic factor in RA, yet information on the likely outcome of RA would help therapeutic decision-making [5]. Although there is a consensus for the efficient control of inflammation at the onset and throughout the course of the disease as the main way to reduce functional decline in RA patients [6], it is unknown whether the functional outcome is influenced by the intensity of initial inflammatory symptoms.

Adjuvant-induced arthritis (AIA) in rats is one of the most widely used animal models of RA and is predictive of the clinical efficacy of many drugs in human RA [7]. With this model, inflammatory symptoms of the first episode of inflammation peak within 2 to 3 weeks after immunization and then progressively resolve. However, even though all immunized rats share a similar time-course of the disease, they differ from each other by the severity of the initial inflammatory symptoms. Therefore, AIA in rats offers the unique opportunity to explore the connection between the severity of initial inflammation and functional outcome. As a hallmark of AIA in rats is bilateral ankle damage [8], locomotion analysis seems to be an appropriate tool to assess motor disabilities. Notably, in previous studies aimed at investigating locomotion in animal models of mono- and polyarthritis, locomotion analysis was restricted to the acute inflammatory period of the disease [9–16].

The present study examined the prognostic value of the severity of the initial inflammation on locomotor outcome in AIA rats. For this purpose, we explored the relationships between locomotor parameters measured at day 195 (approximately 7 months) post-immunization and the arthritis score or hind-paw diameter measured early during the initial inflammatory period. Multiple locomotor parameters including time and length parameters, limb kinematics, lateral paw position at toe-off, maximal hind-paw elevation, posture, and ROM of the ankle were measured using 3D imaging technology. Radiographic images of the hind paws were taken and scored just after the locomotion recording. AIA and age-matched non-AIA rats were studied in parallel.

## Methods

### Animals

Six-week-old male Lewis rats (n = 38) were purchased from Janvier (Le Genest Saint Isle, France). The animals were kept under a 12 h-12 h light:dark cycle and allowed free access to food and water. The experimental procedures were approved by the local committee for ethics in animal experimentation (#0411, date 01/18/2011) of Université de Bourgogne (Dijon, France), and complied with the Animal Research Reporting In vivo Experiments (ARRIVE) guidelines. The same operator performed all steps of the experiments (license number 21CAE035).

### Selection of animals

All the rats were first handled gently for a few days and familiarized with the treadmill apparatus (Bioseb, Vitrolles, France) in order to reduce stress due to novelty. Then, they were selected according to their ability to walk regularly on a horizontal treadmill with the speed of the treadmill belt fixed at 30 cm/s. Three 30-second running sessions were given twice a day for seven days. Mild intensity electric shocks to the feet were used as negative reinforcement to improve performance. Rats that failed to walk in a regular manner on the treadmill (contact of the forelimbs with the front wall of the treadmill, frequent immobility or galloping) at the end of the selection period were excluded from further experiments. Of the 38 Lewis rats enrolled in the study, 32 were able to walk in a regular manner on the treadmill. These rats were divided into AIA rats (n = 23) and control rats (n = 9).

### Induction of arthritis

AIA was induced under volatile anesthesia (halothane) by a single intradermal injection at the base of the tail of 1 mg of heat-killed *Mycobacterium butyricum* (Difco, Detroit, MI, USA) suspended in 0.1 ml of Freund’s incomplete adjuvant (Difco). A group of non-arthritic age-matched rats received an equal volume of saline and were used as control rats. The incidence of arthritis as assessed from clinical scoring reached 91 % (21/23 immunized rats). Rats (n = 2) that did not develop arthritis were excluded from further analysis.

### Assessment of clinical signs of inflammation

The rats were weighed and monitored for clinical signs of arthritis from immunization to week 27 (every two days from day 9 to 80 and every 15 days from day 80 to 195). The clinical scoring system (arthritis score) was employed as follows [17]: one finger scores 0 (no arthritis) or 0.1 (redness or swelling of one finger) and each big joint (ankle or wrist) scores 0 (no arthritis), 0.5 (mild but definite redness and swelling) or 1 (severe redness and intense swelling). The tarsus and ankle was considered the same joint. The arthritis score for a given limb ranged from 0 to 1.5 and the global arthritis score (four limbs) ranged from 0 to 6. The clinical scores were further divided into five grades: grade 0 (arthritis score = 0), grade 1 (arthritis score between 0.1 and 0.9), grade 2 (arthritis score between 1 and 1.9), grade 3 (arthritis score between 2 and 2.9), grade 4 (arthritis score between 3 and 3.9) and grade 5 (arthritis score more than 4). As the arthritis score only provides a subjective quantification of inflammation, it was coupled with the measurement of hind paw diameter using a digital caliper (Fischer Darex, France). The values were expressed as the mean of the two hind paw diameters. When indicated, values for individual hind paw diameters were presented.

### Radiographic analysis of hind paws

Radiographs of hind paws were performed at the end of the experiment (week 27 after immunization) with a BMA High Resolution Digital X-rays machine (40 mV, 10 mA) - D3A Medical Systems (Orléans, France). A global score was determined for each hind paw using a modification of the grading scale described by Esser et al. [18]. This score evaluates both joint degradation, which was assessed from joint space narrowing and erosion, and new bone formation, which was assessed from periostitis and heterotopic bone. Joint degradation was scored at the tibiotalar, tarsal and subtalar joints as follows: normal aspect (0), mild degradation (1), moderate degradation (2) and severe degradation (3). New bone formation was scored at the calcaneum, tibia and tarsal bones as follows: normal aspect (0), mild hyperostosis (1), moderate hyperostosis (2) and severe hyperostosis (3). Radiographs were rated by two independent experienced observers. For each hind-paw, the maximal global radiographic score was 18 while the maximal joint degradation and the maximal new bone formation scores were both 9.

### Locomotion recordings

Locomotion was recorded at week 25, 26 and 27 after immunization and at corresponding times in controls. Data were collected using the VICON MX-13 optical motion capture system (Vicon, Oxford, Great Britain), which consists of six high-speed digital infrared cameras as previously described in detail by our laboratory [19, 20]. Briefly, after anesthesia (intraperitoneal chloral hydrate, 400 mg/kg) the four limbs and the back were shaved and tattooed in order to locate the bony processes. Twenty-two reflective hemispherical markers (BTS Bioengineering, Cod FMK0005, Milano, Italy) with a diameter of 6 mm were placed over the following anatomical landmarks: the scapula, the upper (shoulder marker) and lower (elbow marker) humerus epiphysis, the metacarpophalangeal (MCP) joint, the iliac crest, the great trochanter, the knee, the external malleolus and the fifth metatarsophalangeal (MTP). Four markers (markers 1, 2, 3 and 4) were also placed on the back from the neck to the tail at regular distances. Three arthritic rats and one control rats died from anesthesia while being tattooed, thus, locomotion was recorded in eighteen AIA rats and eight control rats. Locomotion was recorded with the speed of the treadmill belt fixed at 30 cm/s, a speed that is within the range of the locomotion speed of rats over ground [21] and for a 1-minute session without delivering foot shocks. Soft tissue movement around the knee (skin slippage) is a recognized source of error when estimating joint kinematics of hind limbs in rats from markers placed on the surface of the body overlying joints [22]. To investigate this potential error, we measured the variation coefficient of the distance between the knee marker and the external malleolus marker at toe contact and at toe-off in both control and AIA rats.

### Locomotion analysis

The gait cycle (defined as the time between two successive foot contacts of the same limb), was split into two parts, the stance and the swing phase. The stance phase was defined as the part of the cycle that begins when the foot strikes the treadmill belt and terminates when the foot starts its forward movement (i.e., when the vertical velocity of the MTP markers was higher than a threshold fixed at 5 % of its maximal velocity). The swing phase was considered to begin at the onset of forward movement and to end when the foot strikes the treadmill belt. Using a MATLAB custom-program (Math-Works, Natick, MA, USA), we measured the following locomotion-related parameters with a reference frame fixed to the hip marker:

- stance and swing phases duration, and gait cycle duration
- stride length, which was computed as the Euclidian distance (mm) of the more distal markers (MTP for the hind-limbs, MTC for the fore-limbs) throughout the swing phase
- maximal (Max) and minimal (Min) excursion of joint angles (degrees) during the stance and the swing phases
- paw location (mm) of the more distal marker of limbs (MTP or MCP) at toe-off in the frontal plane with respect to the axis passing through the hip for the hind limbs and the shoulder for the fore-limbs. A positive angular value indicates a paw placement further to the side
- maximal paw elevation during the cycle (mm).

These parameters were calculated for each hemibody in both control and AIA rats. For AIA rats, they were calculated from the more and less impaired hemibody (hemibody with the highest and lowest hind paw diameter just before locomotor recording, respectively).

We also calculated the following parameters:

- ROM of lateral roll of the body (degrees). The parameter was assessed from the measurement of the lateral tilt angle between the horizontal plane of the laboratory and the line passing through the two hip markers
- sagittal tilt of the body (degrees). The parameter was assessed from the measurement of the angle between the horizontal plane of the laboratory and the line passing through markers 1 and 4 of the back. A negative angular value indicated elevation of the hindquarter with respect to the head.

Even though all AIA rats were able to walk on the treadmill, periods with irregular locomotor cycles (walking on only three limbs, successive jumps and short periods of immobility followed by increased velocity of walking) were more frequent in AIA than in control rats. In certain AIA rats, the digits of the hind paws were often curled while walking with no contact of the calcaneum with the treadmill belt. From a careful visual inspection of walking AIA rats, we also detected hind-paw eversion. Unlike control rats, AIA rats often used their tail to walk. Finally, the observation of AIA rats in their housing cages revealed that these rats avoided standing on their feet. It is noteworthy that locomotion-related parameters were all calculated from at least four regular and consecutive step cycles during each trial in order to eliminate deviant curves [23].

### Data and statistical analysis

Values are presented as the mean ± standard deviation except for the data on recurrence of inflammatory episodes, which were expressed by the median. Comparisons of locomotion-related parameters between the left hemibody of control rats and the two hemibodies of AIA rats were made using the Kruskall Wallis’ test followed by the Mann-Whitney *t* test and the Bonferroni correction. Differences between the more and less impaired hemibodies in AIA rats were assessed using Wilcoxon’s test for pairwise comparisons. The relationship between two variables was investigated using the Pearson’s correlation coefficient. *P* <0.05 was considered statistically significant.

## Results

### Time course of arthritis scores and hind-paw diameters in AIA

**Fig. 1:**
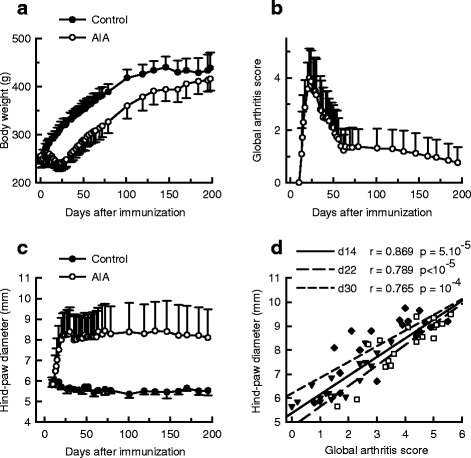
Time course of arthritis. Body weights (**a**), global arthritis scores (**b**), and mean hind-paw diameters (**c**) were measured in control rats (n = 8) and rats with adjuvant-induced arthritis (*AIA*) (n = 18) for 195 days after immunization. **d** In AIA rats, mean hind-paw diameters correlated positively with global arthritis scores at day (*d*) 14, 22 and 30 after immunization. Values are means ± SD

The results are summarized in Fig. 1. Arthritis was associated with an early, long-lasting but reversible loss of body weight (Fig. 1a). The first clinical signs of arthritis were observed as early as day 10 after immunization. At day 14, all AIA rats (n = 18) exhibited signs of inflammation. The mean global arthritis score (Fig. 1b) peaked at day 22 post-immunization (between days 20−24 post-injection for 17 rats). At this time, the distribution of rats according to the arthritis grade was as follows: 56 % with grade 5, 28 % with grade 4, 11 % with grade 3 and 5 % with grade 2. The mean global arthritis score decreased abruptly from day 22 to day 60 and more progressively thereafter. Notably, all the limbs exhibited clinical signs of arthritis even though signs were more intense in the hind limbs than the forelimbs throughout the disease. Hind-paw diameter (mm) in AIA rats was 5.8 ± 0.4 at day 9 (before the first signs of inflammation), increased to 8.0 ± 1.1 at day 20, peaked at day 30 post-immunization (8.4 ± 1.2) and then remained stable (Fig. 1c). By contrast, mean hind-paw diameters in control rats did not change from day 9 to day 195 post-immunization. Of note, the global arthritis score and hind-paw diameter were highly correlated at days 14, 22 and 30 post-immunization, as shown in Fig. 1d. At day 195, the parameters were still correlated (*r* = 0.813, *p* = 2.10^-5^, not shown). Recurrent inflammatory disease (one to five relapses) was observed in most AIA rats (15/18 rats, 83 %) within the period week 6 to week 23 post-immunization, and restricted to the hind limbs. As compared with the first episode of inflammation, inflammatory signs related to relapses were weak and short lasting (3 days). In addition, relapses did not occur at the same time in the population of AIA rats, and as a result relapses could not be detected from the analysis of the time course of the global arthritis score. Notably, and consistent with the lack of a relationship between the severity of the first episode of inflammation and relapse frequency, the number of relapses expressed as a median affected by minimal-maximal values was 2 (0−4) in rats with grade 5 (n = 10), 3 (2−4) in rats with grade 4 (n = 5) and 3 (0−5) in rats with grade 2 or 3 (n = 3) at day 22 post-immunization, i.e., at the peak of the global arthritis score.

### Long-term AIA is associated with impaired locomotor patterns and locomotion kinematics

Only locomotor parameters recorded at week 27 (day 195, approximately 7 months post-immunization) are presented here because no difference was observed between the three recordings either in AIA or in control rats. As visual examination of the hind limbs before the first locomotion recording revealed bilateral but relatively asymmetrical paw deformation in AIA rats, we suspected asymmetrical impairment of locomotion parameters. Therefore, locomotor parameters for the hemibody with the highest and the lowest hind-paw diameter, measured just before the first recording of locomotion, were calculated separately. This diameter (mm) was 9.2 ± 1.8 and 7.3 ± 1.4 for the more and less impaired hind-limb, respectively (*p* <0.05). In control rats, locomotor parameters were calculated from the left hemibody (hind-paw diameter was 5.5 ± 1 mm).

#### Effect of arthritis on timing and length parameters

**Table 1:** Effect of AIA on timing and length parameters

	Control	AIA	
		More impaired hemibody	Less impaired hemibody
Hind-limbs			
Cycle duration (ms)	504 ± 16	485 ± 28	483 ± 29
Stance duration (ms)	376 ± 22	351 ± 34	361 ± 37
Swing duration (ms)	128 ± 12	134 ± 22	122 ± 18
Cycle length (mm)	80 ± 5	76 ± 8	81 ± 9
Fore-limbs			
Cycle duration (ms)	506 ± 18	474 ± 39	473 ± 41^a^
Stance duration (ms)	324 ± 12	322 ± 27	309 ± 30
Swing duration (ms)	182 ± 12	152 ± 23^a^	164 ± 22
Cycle length (mm)	80 ± 8	76 ± 5	81 ± 10

Parameters were measured in control (n = 8) and adjuvant-induced arthritis (*AIA*) rats (n = 18) at day 195 post-immunization. The more and less impaired hemibodies corresponded to hemibodies with the highest and lowest hind-paw diameter just before the locomotion recording, respectively. Values are means ± SD. ^a^Significantly different from control rats (*p* <0.05)

The results are shown in Table 1. Timing and length parameters calculated from the hind limbs did not significantly differ between control and AIA rats whatever the hemibody considered. By contrast, the swing phase of the forelimbs was shorter in AIA than in control rats even though the reduction reached significance only for the more impaired hemibody, thus resulting in a shorter cycle duration of the fore-limbs in AIA rats (−6 % (not statistically significant) for the more impaired hemibody and –6 %, *p* <0.05 for the less impaired hemibody, as compared to control values).

#### Effect of arthritis on joint angles

**Fig. 2:**
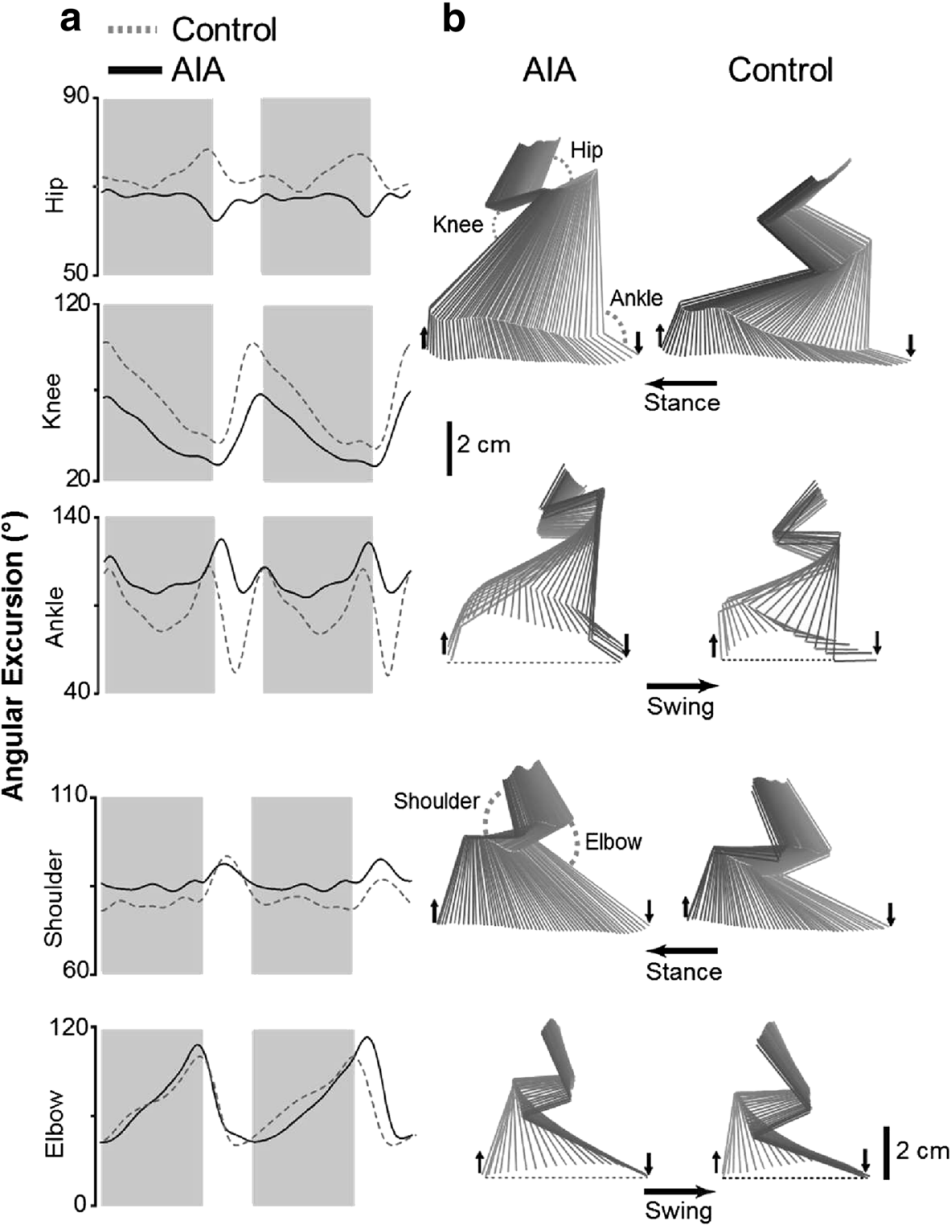
Effect of adjuvant-induced arthritis (*AIA*) on joint angles. **a** Angular excursion: *dashed* and *solid lines* correspond to values measured in representative control and AIA rats at day 195 post-immunization, respectively. The phases of the locomotor cycle were normalized (the stance phase is in gray). **b** Corresponding stick diagrams of one complete step cycle (stance and swing) in control and AIA rats. Horizontal arrows indicate the direction of the movement; *downward arrows* indicate foot contact and *upward arrows* indicate foot lift

In accordance with our expectation that abnormalities in kinematics could be asymmetrical in AIA rats, a difference in joint angles was observed between the more and less impaired hind limb except for the knee (Table 2). The effects of AIA on hind limb kinematics were that the hip and knee were more flexed and the ankle more extended in AIA than in control rats throughout the cycle. The ROM of the ankle was quite different between AIA and control rats during both the stance and swing phase (Table 2). As compared to control values, the ROM of the ankle in AIA rats during the stance phase was decreased by 22 % (not significant) for the less impaired and by 45 % (*p* <0.05) for the more impaired hemibody. The corresponding values during the swing phase were 25 % (not significant) and 55 % (*p* <0.05). Thus, stiffness of the ankles in AIA seemed to compel the rats to keep the joint in extension throughout the gait cycle. As regards the effect of AIA on fore limb kinematics, comparison of the shoulder joint between control and AIA rats suggested that the shoulder was more extended in AIA during both the stance and swing phase. By contrast, no difference was observed between control and AIA rats at the elbow joint. The changes in kinematics induced by arthritis are summarized in Fig. 2, which shows angular excursion (Fig. 2a) and stick diagrams (Fig. 2b) in representative AIA and control rats. Finally, no difference in the variation coefficient of the knee-ankle distance was observed between the hind limbs of control rats or between the less and more impaired hind limbs in AIA rats (not shown), thus indicating that differences between control and AIA rats in values for the knee joint and differences between the hemibodies of AIA rats did not relate to skin slippage.

**Table 2:** Effect of AIA on joint angles

		Control	AIA	
			More impaired hemibody	Less impaired hemibody
Joint angle during the stance phase (°)				
Hip	Min	65.8 ± 9.1	50.4 ± 11.6^a^	57.7 ± 11.9^b^
	Max	76.6 ± 9.1	62.3 ± 11.3^a^	68.7 ± 11.4
Knee	Min	43.4 ± 9.5	42.1 ± 10.6	42.9 ± 11.0
	Max	109.0 ± 14.1	89.8 ± 15.7^a^	92.5 ± 16.0^a^
Ankle	Min	75.9 ± 4.1	100.7 ± 12.5^a^	98.1 ± 12.7^a^
	Max	127.4 ± 8.7	129.0 ± 12.1	138.1 ± 11.5^a,b^
ROM of ankle (°)		51.5 ± 8.0	28.3 ± 11.3^a^	40.0 ± 16.3^b^
Shoulder	Min	68.5 ± 6.7	78.7 ± 9.5^a^	76.2 ± 8.5
	Max	80.6 ± 8.9	93.8 ± 10.3^a^	87.7 ± 9.2^a,b^
Elbow	Min	54.2 ± 9.1	58.1 ± 7.4	52.7 ± 8.4 ^b^
	Max	119.3 ± 9.7	115.7 ± 7.2	114.4 ± 10.7
Joint angles during the swing phase (°)				
Hip	Min	62.3 ± 8.5	45.3 ± 12.0^a^	53.6 ± 12.0^b^
	Max	70.6 ± 8.0	57.7 ± 10.5^a^	64.0 ± 11.3^b^
Knee	Min	41.0 ± 8.8	39.6 ± 8.9	39.8 ± 9.4
	Max	109.0 ± 14.1	89.5 ± 16.0^a^	92.3 ± 16.0^a^
Ankle	Min	60.7 ± 6.2	96.7 ± 15.3^a^	86.5 ± 15.2^a,b^
	Max	112.7 ± 9.1	120.4 ± 12.3	125.7 ± 9.0^a^
ROM of ankle (°)		52.0 ± 7.9	23.6 ± 9.5^a^	39.2 ± 16.4^b^
Shoulder	Min	67.9 ± 7.5	78.0 ± 10.1	74.6 ± 8.2
	Max	81.2 ± 9.8	93.5 ± 10.7^a^	86.6 ± 9.3^b^
Elbow	Min	50.4 ± 8.2	53.7 ± 5.8	48.3 ± 6.8^b^
	Max	118.0 ± 10.9	112.6 ± 8.6	114.0 ± 11.8

Parameters were measured in control (n = 8) and adjuvant-induced arthritis (*AIA*) rats (n = 18) at day 195 post-immunization. The more and less impaired hemibodies corresponded to hemibodies with the highest and lowest hind-paw diameter just before locomotion recording, respectively. Values are means ± SD. ^a^Significantly different from control rats (*p* <0.05). ^b^Significantly different from the more impaired hemibody (*p* <0.05). *ROM* range of motion

#### Effect of arthritis on paw location and posture

**Fig. 3:**
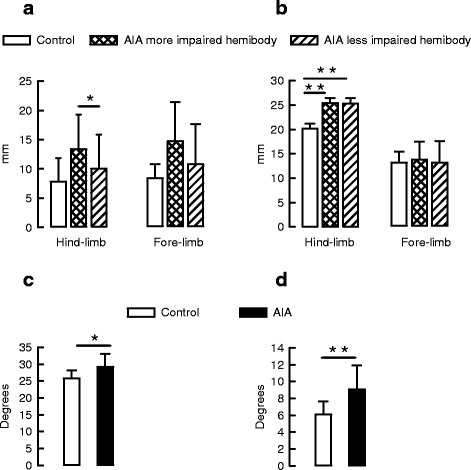
Effect of adjuvant-induced arthritis (*AIA*) on paw location and posture. **a** Lateral paw location of hind-limbs and fore-limbs, **b** maximal paw elevation of hind-limbs and forelimbs, **c** range of motion (*ROM*) of lateral tilt and **d** sagittal tilt were measured in control (n = 8) and AIA rats (n = 18) at day 195 post-immunization. Values are means ± SD: *p <0.05, **p <0.01

The results are summarized in Fig. 3. In control rats, paw location at toe-off in the frontal plane was approximately 8 mm for all limbs (Fig. 3a). Arthritis was associated with trends towards a position further to the side for both fore-limbs and hind-limbs. This abnormality was significantly more pronounced in the more than in the less impaired hind-limb. Maximal paw elevation (Fig. 3b) during the swing phase was significantly higher in AIA than in control rats, at least for the hind-limbs, whatever the hemibody considered. In addition, as shown in Fig. 3c, the ROM of the lateral roll angle was significantly higher in AIA than in control rats, reflecting a greater waddling gait in AIA rats. Finally, the sagittal tilt of the body (Fig. 3d) was significantly higher in AIA than in control rats, indicating that AIA rats walked with a higher position of the hindquarters with respect to the head position.

### Radiographic score reveals joint damage and new bone formation in long-term AIA

**Fig. 4:**
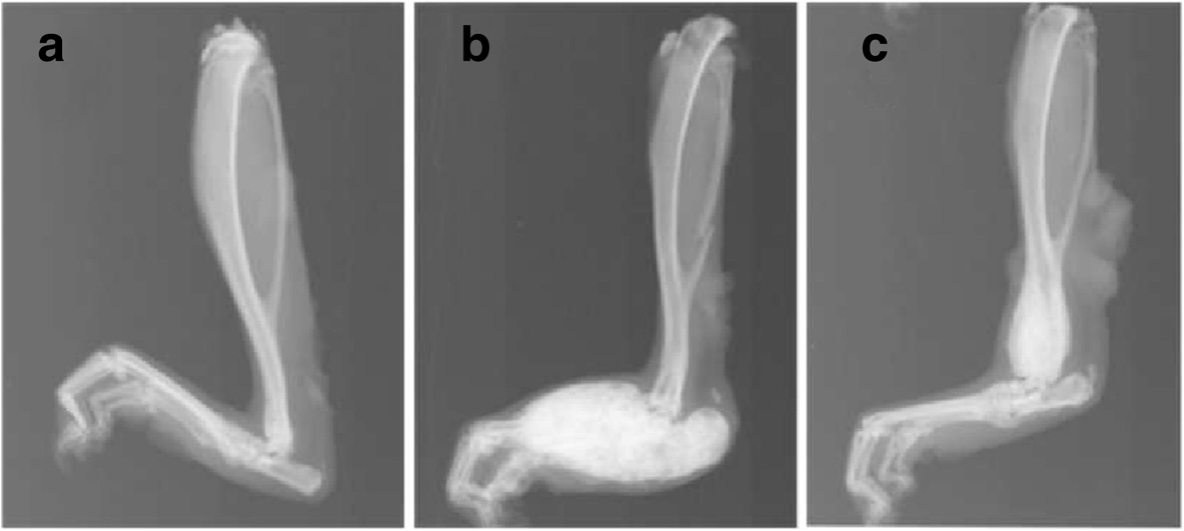
Radiographic changes in long-term adjuvant-induced arthritis (AIA). Representative radiographs of the hind-paw from control rats (**a**) and AIA rats (**b** and **c**) at day 195 post-immunization. Note the severe new bone formation in the tarsal region and calcaneum (**b**) and in the lower extremity of tibia (**c**) in AIA rats

The radiographic score in AIA rats reached 8.6 ± 3.7 for joint degradation and 7.8 ± 3.7 for new bone formation. Fig. 4 illustrates the dramatic dense ossification in the tarsal region, calcaneum or lower extremity of the tibia with destruction inside joints in AIA. The global radiographic score (joint degradation + new bone formation), which was 0.9 ± 0.8 in control rats, reached 10.3 ± 4.1 for the more impaired hemibody and 6.1 ± 4.1 for the opposite hemibody (*p* <0.01, not shown).

**Fig. 5:**
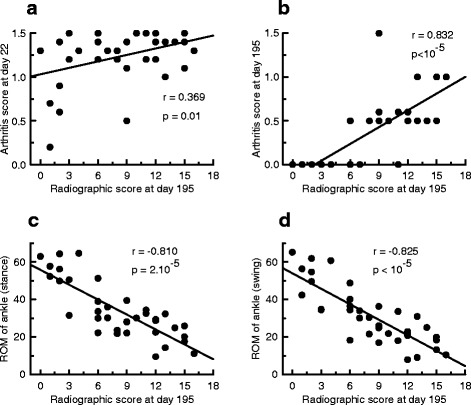
Radiographic score was associated with the arthritis score and range of motion (*ROM*) of the ankle. In rats with adjuvant-induced arthritis (n = 18), global radiographic scores (joint degradation + new bone formation) measured at day 195 were plotted against arthritis scores of corresponding hind-paws at day 22 (**a**) and at day 195 (**b**) post-immunization and against the corresponding ROM of the ankle measured during the stance (**c**) or the swing phase (**d**)

As shown in Fig. 5, the global radiographic score correlated positively with the arthritis score of the corresponding hind paw measured either at day 22 (Fig. 5a) or at day 195 (Fig. 5b) after AIA induction. Likewise, significant correlation was found between the global radiographic score and paw diameter of the corresponding hind-paw measured either at day 22 (*r* = 0.627, *p* = 0.003) or at day 195 (*r* = 0.890, *p* <10^-5^, not shown) after AIA induction. The global radiographic score also correlated negatively with the ROM of the ankle measured either during the stance (Fig. 5c) or the swing phase (Fig. 5d). Notably, this negative correlation was still observed when ROM of ankle was plotted against the joint degradation score (*r* = −0.710 and *r* = −0.727 for the stance and the swing phase, respectively, *p* <10^-5^) and against the new bone formation score (*r* = −0.852 and *r* = −0.858 for the stance and the swing phase, respectively, *p* <10^-5^, not shown).

### Abnormalities in locomotion are associated with reduced ankle mobility

Suspecting an association between reduced ankle mobility and impaired locomotion-related parameters in AIA rats, we plotted the values of each individual ROM of the ankle joint (measured either during the stance or swing phase) against the individual ROM of other joint angles in the corresponding phase of the cycle. The results are summarized in Table 3. During the stance phase, only the ROM at the knee correlated significantly with the ROM at the ankle. By contrast, during the swing phase, the ROM at the ankle was correlated negatively with the ROM at the hip, but was positively correlated with the ROM at the knee and the ROM at the elbow. No association was found between the ROM at the ankle and the ROM at the shoulder. The ROM of the ankle either during the stance or the swing phase correlated negatively with maximal hind-paw elevation and lateral hind-paw location at toe off while it correlated positively with the duration of the stance phase for the hind-limbs.

**Table 3:** Correlation between the ROM of the ankle joint and other locomotion-related parameters

	ROM of ankle joint			
	During the stance phase		During the swing phase	
	*r*	*p*	*r*	*p*
ROM of hip joint (°)	−0.176	0.152	−0.288	0.044
ROM of knee joint (°)	0.355	0.017	0.341	0.021
ROM of shoulder joint (°)	−0.277	0.051	−0.212	0.107
ROM of elbow joint (°)	0.243	0.077	0.386	0.010
Maximal hind-paw elevation (mm)	−0.454	0.003	−0.443	0.003
Lateral hind-paw location (mm)	−0.398	0.008	−0.440	0.004
Hind-limb stance duration (ms)	0.387	0.010	0.308	0.034

Parameters were measured in rats with adjuvant-induced arthritis (n = 18) at day 195 post-immunization. Values are means ± SD. *ROM* range of motion, *r* Pearson’s correlation coefficient

### Reduced ankle mobility was associated with intensity of initial inflammation

**Fig. 6:**
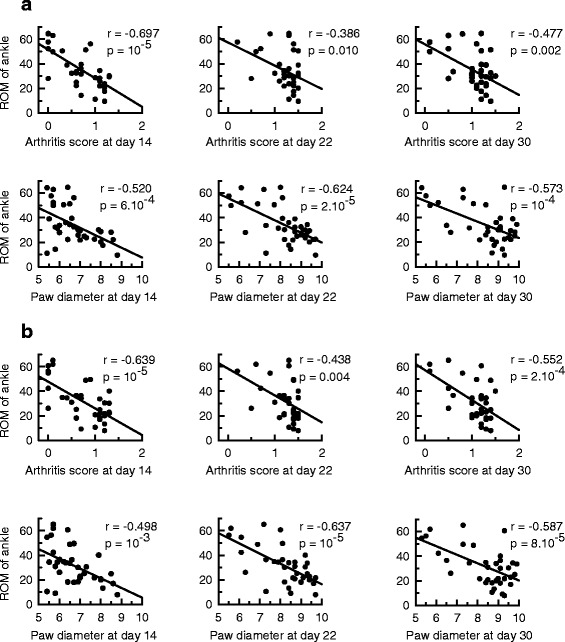
The range of motion (*ROM*) of the ankle was associated with the severity of the first signs of inflammation. In rats with adjuvant-induced arthritis the individual values of the ROM of the ankle at day 195 post-immunization were plotted against individual arthritis scores or hind-paw diameters of the corresponding hind-paws measured at day 14, 22 and 30 post-immunization. The ROM of the ankle was measured during the stance (**a**) and the swing phase (**b**)

To assess the prognostic value of the intensity of initial inflammation on long-term locomotor disability, we explored the association between the arthritis score or hind-paw diameter of each hind-limb at days 14, 22 and 30 post-immunization and the corresponding ROM of the ankle measured at day 195 during both the stance and swing phase (Fig. 6). During the stance phase (Fig. 6a), the arthritis score at days 14, 22 and 30 (upper graph) correlated negatively with the ROM of the ankle. A negative correlation was also found between hind-paw diameter at days 14, 22 and 30 and the ROM of the ankle (bottom graph). Similar results were obtained for the swing phase (Fig. 6b).

## Discussion

Using the AIA model, the present study revealed a strong association between the intensity of initial symptoms of inflammation and the locomotor parameters measured at day 195 post-immunization. It also found a negative correlation between ankle mobility and concurrent hind paw radiographic lesion severity.

Unlike previous studies on AIA rats, which mainly focused on the acute period after immunization, the present study is the first to perform long-term follow-up of the disease in AIA rats (from immunization up to 7 months). Contrary to the general belief that the AIA model is monophasic [7], but consistent with arthritis flares in RA patients, our results revealed that 83 % of rats demonstrated recurrent disease. However, as relapses did not occur at the same time (from week 6 to week 23) in the population of AIA rats, analysis of the time course of the global arthritis score failed to detect them. Moreover, evidence of strong positive correlations between the global arthritis score (calculated from the four paws) and hind-paw diameter (calculated from the two hind-limbs only) at day 14 (onset of inflammation), day 22 (peak of inflammation) and day 30 (onset of inflammation resolution) advocate the use of hind-paw diameter as an objective and rapid quantification of clinical inflammation in AIA rats, at least in the acute period after immunization. Thereafter, though the global arthritis score progressively decreased, hind-paw diameter remained elevated. Thus, hind-paw diameter during and beyond the resolution of the first inflammatory episode has to be seen as an index of new bone formation. A strong positive association was consistently observed between the radiographic score and concurrent hind-paw diameter at day 195 post-immunization. Bone proliferation and the tendency for joint ankylosis in AIA rats were previously reported at 4 months post-immunization [24]. However, these data have gained little attention as new bone formation is not a feature of RA. By contrast osteoproliferation is frequent in spondyloarthritic patients, suggesting that AIA in its late phase could be a relevant model of structural damage associated with spondyloarthritis.

The present study provides for the first time a comprehensive analysis of locomotion in an animal model of RA. It clearly identified reduced ankle mobility as a driver of locomotor abnormalities (Table 3). These data are in line with clinical studies that reported the involvement of impaired mobility of the foot-ankle complex in the walking disability of RA patients [1, 25–28]. Although it was not in the scope of our study to explain the mechanisms involved in AIA-induced locomotor impairment, the following scenario could be surmised. The limitation of plantar flexion during the swing phase forces AIA rats to further elevate their hind-paws to prevent their contact with the treadmill belt and subsequent stumbles. This adaptive strategy compromises balance thereby activating compensatory strategies including the use of the tail as an additional limb and a wider position of the limbs at toe-off. The exaggerated sagittal tilt of the body during locomotion could be interpreted as a postural change to shift body weight towards the fore-limbs in order to reduce weight-bearing on painful ankles. Likewise, the negative correlation between the ROM of the ankle and stance phase duration supports the idea that AIA rats try to alleviate the compressive force applied to the damaged ankles. Finally, evidence of a waddling gait in AIA rats may reflect exaggerated hind paw elevation during the swing phase and accompanying changes in pelvic girdle mobility.

Factors associated with walking disability in RA patients in remission have been poorly studied. Pain, limited joint ROM, loss of functional balance or muscle loss are all thought to contribute to walking disability in RA [1–4]. Consistent with a link between evidence of damage on x-ray and functional disability in late RA [28–30], our results demonstrated strong correlation between joint degradation and concurrent ROM of the ankle in AIA rats. The new finding is that ankle mobility measured in the late phase of AIA correlated negatively with the arthritis score and with the hind-paw diameter when measured during the first inflammatory episode, thus, highlighting the predictive value of early clinical inflammation for long-lasting problems in arthritis-associated locomotion. These correlations were observed not only at the peak of inflammation (day 22) but also at the onset of clinical symptoms (day 14).

These data have both therapeutic and methodological perspectives. From a therapeutic perspective, they suggest that the intensity of initial symptoms of inflammation may provide information on the likely motor outcome in RA patients. In these conditions, the efficient control of inflammation as early as possible in RA patients is expected to be the best way to prevent motor disabilities. As conventional or biological disease-modifying anti-rheumatic drugs (DMARDs) cannot be initiated before RA diagnosis, the early blockade of inflammation can be achieved only with anti-inflammatory drugs. In agreement with the use of corticoids to deal with the first signs of inflammation, the early administration of corticoids decreases inflammatory symptoms in animal models of RA [31, 32]. However, further animal studies are needed to investigate whether early corticotherapy at a dosage able to treat clinical symptoms of inflammation translates into positive effects on long-term locomotor outcome.

Our results clearly emphasize the importance of early anti-inflammatory therapy to prevent locomotor problems associated with arthritis. However, physical disabilities, including decreased locomotor activity, remain a complaint in RA patients in remission. These patients might benefit from the development of strategies that target processes other than inflammation. Among these strategies, there is an increasing interest in physical exercise (which targets neuromuscular plasticity) [33, 34], drugs able to repair joint damage (which target osteoblast/osteoclast imbalance) [35] and glucocorticoids at dosage levels too low to affect the clinical inflammation itself but with a joint-protective effect (unknown targets) [36]. Our results provide methodological perspectives for screening the efficacy of these strategies on motor function in AIA rats. Such strategies need to be initiated after resolution of the acute period of inflammation (to mimic remission in RA patients). As the intensity of the initial inflammation has a predictive value for long-term locomotor outcomes in AIA rats, a simple measurement of paw diameter at the onset of inflammation will allow the early selection of rats with respect to locomotor outcomes. This procedure of selection would increase intra-group homogeneity before randomization of AIA rats into treated and untreated groups and would provide the possibility to reveal significant effects of treatments on locomotor disability.

## Conclusion

The present study is the first to carry out long-term follow up of clinical signs of inflammation and provide a comprehensive analysis of locomotion at the late phase of the widely used AIA model. The results demonstrate the predictive value of the intensity of the first inflammatory event for long-term locomotor outcome.

## Abbreviations

AIA: adjuvant-induced arthritis
MCP: metacarpophalangeal
MTP: metatarsophalangeal
RA: rheumatoid arthritis
ROM: range of motion
